# Herbal Interaction With Chemotherapeutic Drugs—A Focus on Clinically Significant Findings

**DOI:** 10.3389/fonc.2019.01356

**Published:** 2019-12-03

**Authors:** Pius S. Fasinu, Gloria K. Rapp

**Affiliations:** Department of Pharmaceutical Sciences, College of Pharmacy & Health Sciences, Campbell University, Buies Creek, NC, United States

**Keywords:** cancer, chemotherapy, complementary and alternative medicine, drug interaction, herb-drug interaction, pharmacokinetics

## Abstract

One of the most consequential risks associated with the concomitant use of herbal products and chemotherapeutic agents is herb-drug interactions. The risk is higher in patients with chronic conditions taking multiple medications. Herb-drug interaction is particularly undesirable in cancer management because of the precipitous dose-effect relationship and toxicity of chemotherapeutic agents. The most common mechanism of herb-drug interaction is the herbal-mediated inhibition and/or induction of drug-metabolizing enzymes (DME) and/or transport proteins leading to the alteration in the pharmacokinetic disposition of the victim drug. Most mechanistic research has focused on laboratory-based studies, determining the effects of herbal products on DMEs and extrapolating findings to predict clinical relevance; however, not all DME/transporter protein inhibition/induction results in clinical herb-drug interaction. This study reviews relevant literature and identified six herbal products namely echinacea, garlic, ginseng, grapefruit juice, milk thistle, and St John's wort, which have shown interactions with chemotherapeutic agents in humans. This focus on clinically significant herb-drug interaction, should be of interest to the public including practitioners, researchers, and consumers of cancer chemotherapy.

## Introduction

Like regular synthetic and natural drugs, phytochemicals are capable of altering physiologic processes and eliciting toxicity. Despite the scarcity of information on the safety or otherwise of herbal preparations, sales and use of medicinal herbs and complementary medicines have increased globally. In the United States, the passage of the Dietary Supplement Health and Education Act 25 years ago is believed to have further popularized herbal products and enhanced public confidence in the quality of commercial supplements. One of the major concerns in herbal supplementation is the concurrent use with prescription medicine. Based on the study conducted by Rashrash et al. (1) which relied on the data from the 2015 National Consumer Survey on the Medication Experience and Pharmacists' Roles, the practice of combining prescription medicine with herbal supplements among adults in the United States cuts across all disease states, with 38% of prescription drug users reporting concomitant use of herbal products. One of the most frequent users of herbal medicines, according to the study, are cancer patients (43.1%) surpassed only by stroke patients (48.7%). One study reported a 78% prevalence of herbal and supplementary medicine use among patients on chemotherapy, with 27% assessed to be at a risk of deleterious herb-drug interaction (2). In another recent study, more than half of the respondents reported usage of dietary supplements (which include herbal products) along with chemotherapeutic agents (3).

**Figure 1:**
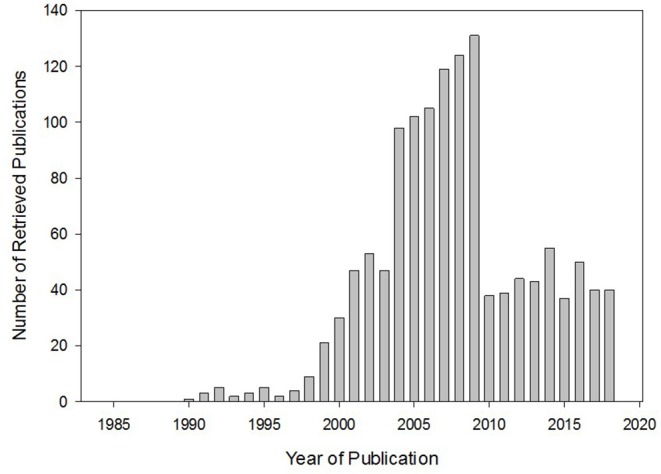
Relevant publications retrieved from PubMed search using “herb-drug interaction” as a search term. The trend shows the introduction of and enhanced interest in herb-drug interaction. Interest has been maintained in this area over the years.

While the benefit of concomitant herb-drug use may be uncertain, one of the known major clinical consequences of such practice is herb-drug interactions. Not well-known until the accidental discovery of the grapefruit juice-felodipine interaction, leading to a 2.8-fold increase in the oral bioavailability of felodipine (4), herb-drug interaction has become an important consideration in pharmacotherapy and is assuming a subcategory of research study on its own. A casual PubMed search with “herb-drug interaction” as a search term would yield no relevant result until after this grapefruit-felodipine phenomenon. Subsequently, the number of herb-drug interaction -related publications increased dramatically, remaining steady over the years (Figure 1) and leading to the introduction of herb-drug interaction as a Medical Subject Heading (MeSH) in 2004.

Herb-drug interactions occur when the pharmacological disposition and/or effect of a drug of interest is altered by the presence of a concurrently administered herbal product. In most cases, herb-drug interactions are mild and could be inconsequential. However, in several instances, therapeutic interventions have been warranted consequent to herb-drug interaction. Such herb-drug interactions include reported bleeding induced by garlic (*Allium sativum*) combined with warfarin, extrapyramidal effects precipitated by betel nuts (*Areca catechu*) in patients taking neuroleptic drugs, and induction of mania in patients taking antidepressants along with ginseng (*Panax ginseng*), among several other clinically significant reported herb-drug interactions (5–7).

Chemotherapeutic agents are generally toxic with an array of side effects. Some of the principal reasons cancer patients combine herbal products with their anti-cancer drugs are the need to manage the side effects associated with chemotherapy and to enhance a general well-being. The potential risk of herb-drug interaction from such herbal use outweighs any benefit. There are several reasons why herb-drug interactions are undesirable in chemotherapy. First, most chemotherapeutic agents have a narrow therapeutic window, thus any alteration in this steep dose-response relationship can lead to toxic manifestations (8). Secondly, plasma concentrations of some chemotherapeutic agents have been shown to be a poor predictor of safety and efficacy (9). Reliance on PK profile in dosage designs have shown a wide inter-individual variation in responses to chemotherapy (10). This is compounded by the variations in the measurable drug concentration in the plasma and the target sites of action. It is thus plausible that slight alteration in the disposition of a chemotherapeutic agent following a delicately established effective and safe dosing will not only be counter-productive but will lead to therapy failure or toxicity. Thirdly, some chemotherapeutic agents, such as ifosfamide and cyclophosphamide, are prodrugs whose efficacy depends on effective biotransformation by cytochrome P450 (CYP) enzymes. Since most herb-drug interactions result from the inhibitory/inductive effect of phytochemicals on these metabolic enzymes, such drugs can easily be rendered ineffective or toxic by herb-drug interaction. Finally, most cancer patients have co-morbidities necessitating the use of multiple drugs, aside from antiemetic agents and other chemotherapy-associated medications. This would increase their risk of experiencing an herb-drug interaction.

Some epidemiological studies have reported that about one-tenth of all general hospital admissions might be due to the effect of multiple drug use resulting in adverse drug interactions and reactions (11, 12). Drug interactions alter drug concentrations in the body, which is particularly undesirable with chemotherapeutic agents that are dosed close to their maximal tolerable levels. On one hand, drug interactions resulting in increased clearance of the cytotoxic drug can lead to subtherapeutic drug exposure, enhance the development of drug resistance, and/or lead to therapy failure. On the other hand, accumulation of cytotoxic drugs resulting from drug interactions can precipitate potentially life-threatening toxicities due to supratherapeutic drug concentrations. Cancer patients often take several medications concomitantly due to co-morbidities and other cancer-associated conditions. In addition to the high risk of drug-drug interactions in such patients, the use of herbal products and the additional risk of herb-drug interaction complicate therapeutic expectations. Finally, the inherent pharmacodynamic effects of the herbal products, including organ-specific effects, and long-term interactions with physiologic receptors may not be beneficial to cancer patients.

Several herbal products have been studied in different patient groups to assess for herb-drug interaction. Clinically relevant information on herb-drug interaction in oncology is generally sparse. Most predictions are based on *in vitro* and preclinical animal studies; however, a few case reports and studies in humans are available to provide perspectives on the risk of herb-drug interactions in clinical settings. Therefore, the aim of this paper is to provide a review of the currently available literature evidence of herb-drug interaction in oncology, with emphasis on herbal products that have shown such interactions in human studies.

## Methods

This is a review conducted to provide an overview of herbal products capable of inducing clinically consequential herb-drug interaction in cancer chemotherapy. The review was systematically conducted by searching PubMed, Medline, Cochrane, Web of Knowledge, Scopus, and Google Scholar databases for original research, and case reports on herb-drug interaction using relevant search terms and the combinations thereof, including common herbal products, individual chemotherapeutic agents, “herbal interactions,” and “herb-drug interactions.” The reference lists of retrieved review papers/meta-analyses were also used to identify relevant publications. Inclusion was limited to publications available in English language and of studies performed in humans to evaluate interactions between herbal supplements and anti-cancer drugs. Searches were not limited by dates or place of publications.

## Results

A total of 345 publications were retrieved. The titles and abstracts were reviewed to determine if publications met inclusion criteria, and only 11 publications met the inclusion criteria. All of the databases searched, except Cochrane, returned the 11 clinically relevant studies. Cochrane did not have the clinical case reports. The included studies covered six herbal products—echinacea, garlic, ginseng, grapefruit juice, milk thistle, and St John's wort—which have been investigated in humans for potential interaction with chemotherapeutic agents. A summary of these studies is provided in Table 1. Subsequent subsections discuss these results. A highlight of the applicable mechanism of herb-drug interaction in cancer chemotherapy was also extracted and discussed below.

**Table 1:** Studies of herbal interaction with chemotherapeutic agents conducted in human subjects.

**Herbal product**	**Cancer drug**	**Study type and description**	**Findings**	**References**
Echinacea	Etoposide	Case report	Taking echinacea with etoposide was found to significantly decrease the platelet nadir (16 × 10^3^/L) when compared to the nadir of etoposide alone (44 × 10^3^/L)	(13)
Echinecea	Docetaxel	Prospective study in 10 cancer patients	Echinacea did not cause significant alteration in the pharmacokinetics of docetaxel	(14)
Garlic	Docetaxel	Prospective, patient controlled, pharmacokinetic	Garlic was found to decrease docetaxel clearance. Although this decrease was non-statistically significant, it could potentially increase adverse effects due to accumulation of docetaxel	(15)
Ginseng	Imatinib	Case report	Patient taking imatinib for 7 years started having symptoms of hepatotoxicity after beginning to consume ginseng. Hepatotoxicity resolved upon discontinuation of ginseng	(16)
Grapefruit juice	Docetaxel	Case report	Grapefruit juice was found to increase the AUC and terminal half-life of docetaxel, while decreasing clearance of docetaxel	(17)
Grapefruit juice	Nilotinib	Open label, randomized, 2 period crossover	Grapefruit juice was found to increase the AUC and peak concentration of nilotinib but did not affect the elimination half-life	(18)
Milk thistle	Irinotecan	Pharmacokinetic study	Milk thistle was found to cause a statistically insignificant decrease in irinotecan clearance, making it unlikely to cause a clinical impact	(19)
St John's wort	Docetaxel	Pharmacokinetic study	St John's wort was found to cause a significant decrease in plasma docetaxel concentration	(20)
St John's wort	Irinotecan	Unblinded, randomized crossover study	St John's wort caused a decrease in plasma concentrations of active metabolite (SN-38) by 42%	(21)
St John's wort	Imatinib	Open label, crossover pharmacokinetic study	St John's wort decreased plasma concentration of imatinib by 32% and decreased the half-life of imatinib by 21%	(22)
St John's wort	Imatinib	2 period, open-label, fixed sequence study	St John's wort increased clearance of imatinib by 43%, and decreased its plasma concentration by 30%	(23)

### Applicable Mechanisms of Herb-Drug Interaction in Oncology

Understanding the mechanism of herb-drug interaction can help predict potentially harmful interactions. The mechanism of herb-drug interaction can broadly be categorized as pharmaceutical, pharmacodynamic, and pharmacokinetic. Pharmaceutical interactions usually arise from physicochemical incompatibility among drugs and formulations when they come in close proximity, such as in an IV bag. Information on pharmaceutical incompatibility of the various herbal formulations with prescription drugs is generally non-existent. This type of interaction is also not very likely with herbal products because little to no contact often exist with prescription drugs before any concomitant administration. Pharmacodynamic interactions are those involving the potentiation, additive, or antagonistic effect of a drug by the presence of an herbal product. To predict this, the biomolecular and pharmacological effect of the individual herbs and their phytoconstituents must be understood. Very little is known about the identity and biological effect of the active phytochemicals in the myriad other herbs used by patients that are not discussed here. However, the potential for pharmacodynamic herb-drug interaction is always present due to the ability of phytochemicals to interact with biological receptors. For example, the antidepressant effect of St John's wort may be expected to be additive in patients taking prescription drugs for the treatment of depression.

The most important category of herb-drug interaction has been identified as pharmacokinetic. The majority of clinically significant pharmacokinetic drug interactions occurs due to the inhibition or induction of the metabolism/clearance of one drug by another (24). This is molecularly mediated by drug metabolizing enzymes and transport proteins. Most anti-cancer drugs are substrates of CYPs and transport proteins (Table 2). Phytochemical compounds are capable of inhibiting and/or inducing drug-metabolizing enzymes, particularly the CYPs. CYP inhibition delays the clearance of CYP substrates, leading to drug accumulation. This is undesirable in cancer chemotherapy due to the narrow therapeutic window of many anti-cancer drugs. CYP inhibition is also deleterious for CYP-dependent prodrugs like ifosfamide and cyclophosphamide, whose biotransformation, once stalled, can lead to therapy failure. The induction of CYP enzymes lead to increased metabolic activity and reduced drug exposures. The resultant sub-therapeutic exposure can lead to treatment failure in the short term, and drug resistance in the long term. Enzyme inhibition/induction affect both the bioavailability and clearance of cancer drugs. Several herbal products including St John's wort, ginkgo, ginseng, licorice, kava, garlic, cranberry, grape seed, germander, goldenseal, valerian, and black cohosh, among others have been shown to inhibit or induce CYPs (24, 25). Similar inhibitory and inductive effects of herbal products on phase II enzymes have been variously reported (26–29). There can also be inhibition/induction of renal excretion and alteration of tissue distribution through displacement from protein binding.

**Table 2:** Several anti-cancer drugs are substrates of drug-metabolizing enzymes and transport proteins.

**Metabolizing enzyme/transporter**	**Anti-cancer substrates**
CYP1A1/1A2	Axitinib, bendamustine, bortezomib, dacarbazine, etoposide, exemestane, flutamide, pazopanib, pomalidomide, tegafur
CYP2A6	Cyclophosphamide, ifosfamide, letrozole, tegafur
CYP2B6	Busulfan, cyclophosphamide, docetaxel, doxorubicin, ifosfamide, procarbazine, thiotepa
CYP2C8	Anastrozole, dabrafenib, cyclophosphamide, enzalutamide, ifosfamide, imatinib, lapatinib, nilotinib, paclitaxel, pazopanib, tegafur
CYP2C9	Busulfan, ifosfamide, idarubicin, ruxolitinib, tamoxifen
CYP2C19	Axitinib, bortezomib, cyclophosphamide, ifosfamide, lapatinib, pomalidomide, tamoxifen, thalidomide
CYP2D6	Brentuximab, doxorubicin, gefetinib, idarubicin, pomalidomide, tamoxifen, vinblastine, vinorelbine
CYP2E1	Dacarbazine, etoposide, cisplatin, vinorelbine
CYP3A4/3A5	Anastrozole, axitinib, bortezomib, bositinib, brentuximab, cabazitaxel, cisplatin, crizotinib, cyclophosphamide, dabrafenib, dasatinib, docetaxel, doxorubicin, enzalutamide, etoposide, exemestane, gefetinib, imatinib, fulvestrant, ifosfamide, irinotecan, lapatinib, letrozole, mitoxantrone, nilotinib, olaparib, paclitaxel, pazopanib, pomalidomide, ponatinib, procarbazine, regorafenib, ruxolitinib, sorafenib, sunitinib, temsirolimus, teniposide, thiotepa, topotecan, trabectedin, vandetanib, vemurafenib, vinblastine, vincristine, vinorelbine
GSTs	Busulfan, carboplatin, chlorambucil, cisplatin, cyclophosphamide, dactinomycin, daunorubicin, doxorubicin, etoposide, idarubicin, ifosfamide, mitomycin, mitoxantrone, oxaliplatin, tamoxifen, vinblastine, vincristine, vinorelbine
UGTs	Anastrozole, axitinib, bicalutamide, doxorubicin, epirubicin, etoposide, exemestane, irinotecan, sorafenib, regorafenib, tamoxifen, teniposide, topotecan
P-glycoprotein (ABCB-1, MDR-1)	Axitinib, bicalutamide, bosutinib, cytarabine, dactinomycin, dasatinib, daunorubicin, docetaxel, doxorubicin, epirubicin, etoposide, gefetinib, idarubicin, imatinib, irinotecan, methotrexate, mitoxantrone, paclitaxel, sunitinib, vincristine
MRP-1 (ABCC-1)	Chlorambucil, daunorubicin, doxorubicin, epirubicin, etoposide, idarubicin, irinotecan, melphalan, methotrexate, mitoxantrone, tenoposide, topotecan, vinblastine, vincristine
MRP-2 (ABCC-2)	Methotrexate, sulfinpyrazone, vinblastine
BCRP (ABCG-2, MXR)	Bicalutamide, dasatinib, docetaxel, daunorubicin, doxorubicin, epirubicin, gefetinib, idarubicin, imatinib, irinotecan, mitoxantrone, nilotinib, paclitaxel, sorafenib, sunitinib, topotecan

*ABC, ATP-binding cassette; BCRP, breast cancer resistant protein; MDR, multidrug resistance gene; MRP, multidrug resistance-associated protein; MXR, mitoxantrone resistance-associated protein*.

Pharmacokinetic herb-drug interactions are also mediated by herbal interaction with transport proteins, principal among which is P-glycoprotein (P-gp). P-gp, also referred to as the multidrug resistance protein 1 (MDR1), or ATP-binding cassette sub-family B member 1 (ABCB1), is a 160-kD ATP-dependent efflux surface glycoprotein first identified in Chinese hamster ovary cells (30). P-gp is localized in various tumors expressing the MDR phenotype. In normal cells, P-gp is expressed in the apical or luminal membranes of cells with excretory or barrier functions including the liver, kidney, intestines, and adrenal glands. P-gp is also a principal constituent of the physiologic blood-brain, blood-testes, and blood-ovary barriers. These anatomical and physiological positions of P-gp enhances its protective and detoxifying functions. In relation to drugs and other xenobiotics, the efflux activity of P-gp reduces cellular penetration and tissue distribution.

As a high-capacity transport protein, the activity of P-gp affects a wide range of structurally unrelated and pharmacologically diverse drugs, including chemotherapeutic agents, anti-retroviral drugs, immunosuppressants, cardio-active drugs, centrally-acting drugs, and several others. Numerous other drugs inhibit the activity of P-gp. Notable among these are verapamil and cyclosporine, used as standard controls in P-gp studies. Many other drugs, including ketoconazole, quinidine, ritonavir, etc., have caused adverse drug interactions through their inhibitory activity on P-gp. Herbal products and phytochemicals including silymarin and extracts from milk thistle, ginseng-derived ginsenosides, piperine, capsaicin, and several others have been reported to inhibit the activity of P-gp. Both the expression and activity of P-gp, like CYPs, can be induced (31–34). St John's wort is an example of a typical herbal P-gp inducer.

### Herbal Products That Have Shown Clinical Interactions With Chemotherapeutic Drugs

#### Echinacea

Formulations of echinacea are globally popular for complementary treatment of respiratory infections and common cold. Among cancer patients, echinacea is popular as an immunomodulatory supplement (35, 36). Recent studies in animals have suggested that echinacea may have beneficial effect in abating some forms of cancer, like leukemia (37). The active constituents and the pharmacological mechanism of any beneficial effect is poorly understood. Echinacea is ranked one of the top widely sold herbal preparations in the United States (38). Most of the preparations of echinacea in the United States are made from one out of the nine common species—*Echinacea purpurea*. Several pre-clinical studies have suggested herb-drug interaction between echinacea and anti-cancer drugs. For example, extracts of echinacea induce P-gp and CYP3A4, two major enzyme/transporter combination that play major roles in the biotransformation and pharmacokinetics of anticancer drugs [Table 1; (39)]. Echinacea is also an inhibitor of CYP3A4 (40). This dual ability to inhibit and induce drug-metabolizing enzymes makes it difficult to predict clinically significant herb-drug interaction with the various CYP/P-gp drug substrates. In human studies, echinacea caused significant increase (34%) in the systemic clearance of midazolam, a CYP3A4 substrate (41). Therefore, there is a potential for herb-drug interaction between echinacea and anti-cancer drugs.

While preclinical studies have shown strong evidence of echinacea interacting with CYP and transport proteins, there is insufficient clinical data on herb-drug interaction with anti-cancer drugs. In a study in 10 cancer patients, echinacea did not cause significant alterations in the pharmacokinetics of docetaxel, which is a substrate of CYP3A4 and P-gp (14). The patients received an intravenous dose of docetaxel on day 1, and were then treated with echinacea supplementation (20 oral drops three times daily of a commercially available product) on days 7–21. They were then administered with another dose of docetaxel on day 22. No significant changes were observed in the pharmacokinetics of docetaxel with or without echinacea supplementation. However, with darunavir, an antiretroviral drug, echinacea caused a general decrease in concentration in the HIV/AIDS patient participants (42).

In a case report, echinacea caused a significant interaction in a cancer patient taking etoposide (13). The adult patient, who was newly diagnosed with squamous cell carcinoma of the lung received cisplatin and etoposide on the first day of treatment with a recorded normal bloodwork. However, by day 8 of this first cycle chemotherapy, his platelet count had dropped by over two-thirds, necessitating platelet transfusion. The discontinuation of echinacea in the cycle 2 chemotherapy helped the patient avoid any further need for platelet transfusion. No further incidence was reported until discharge after 20 days in the hospital. Patient was instructed to avoid taking any more herbal supplements. Etoposide, a cytotoxic agent, is a CYP substrate, whose dose-limiting toxicity is myelosuppression. This interaction is understood to have been as a result of echinacea-induced CYP inhibition, leading to etoposide accumulation and the resultant thrombocytopenia.

#### Garlic

Garlic (*Allium sativum*) is one of the most popular herbal products used to supplement the treatment of infection, diabetes, and heart diseases (43). Its use is common among people with chronic diseases, such as cancer. The major bioactive component of garlic is allicin (diallyl thiosulfinate). Whole garlic extracts have been shown to inhibit the CYP3A4-dependent formation of 6β-hydroxytestosterone from testosterone through *in vitro* liver microsomal incubations (44).

In a study to assess the effect of garlic supplementation on the pharmacokinetics of docetaxel, Cox and co-workers administered docetaxel to women with metastatic breast cancer weekly for 3–4 weeks. A 12-day supplementation with twice-daily 600 mg garlic was commenced on the participants 3 days after the initial dose of docetaxel (15). By Day 15 of the study, garlic supplementation reduced the clearance of docetaxel by 36% (from 30.8 to 20.0 L/h/m^2^. Although, changes in the other pharmacokinetic parameters were reported to be insignificant, the decrease in docetaxel clearance in the presence of garlic may pose significant risk of toxicity due to docetaxel accumulation. This interaction is also consistent with the ability of the phytochemicals in garlic to inhibit CYP enzymes, which are responsible for the metabolism of docetaxel.

#### Ginseng

Ginseng is one of the most popular herbal products sold globally and especially in the United States. Commercial ginseng products are made mainly from three of the several species of ginseng—*Panax ginseng* (Asian ginseng), *Panax quinquefolius* (American ginseng), and *Panax japonicus* (Japanese ginseng). Most therapeutic claims including energy boosting, immunomodulation, enhancement of sexual desire, and pain management are anecdotal. Pharmacological activity of ginseng is generally attributed to ginsenosides, a group of steroidal saponins, which forms the primary phytochemical constituents. Anti-oxidant and cardiovascular protective effect of ginseng have been reported (45, 46). Other reported pharmacological activity of ginseng include immunomodulatory and anticarcinogenic effects, neurotransmitter modulation, and antimitogenic activity (47–49).

There have been mixed findings on the effect of ginseng on drug-metabolizing enzymes and P-gp. In *in vitro* studies, some studies reported no inhibitory activity on CYPs, contrary to others which found inhibitory activity against DMEs (50–54). In a study involving eight healthy volunteers, the effect of the extracts of *P. ginseng* on the pharmacokinetics of midazolam and fexofenadine—substrates of CYP3A4 and P-gp, respectively, was evaluated. Results showed a significant reduction in the AUC and C_max_ of midazolam, which the authors attributed to the inductive effect of ginseng on CYP3A4/5 (55).

As a popular herbal supplement among cancer patients, ginseng has the potential to mediate clinically significant interactions with chemotherapeutic agents. In a case report, an onset of imatinib-induced hepatoxicity was reported in a patient who was being treated with imatinib for chronic myelogenous leukemia (CML). Having used imatinib for 7 years, the patient developed liver dysfunction (confirmed by abnormal liver function test results showing elevated alanine aminotransferase, aspartate aminotransferase, alkaline phosphatase, total bilirubin, and albumin; as well as liver biopsy) only after concurrent use with a *P. ginseng*-containing energy drink for 3 months (16). The symptoms of hepatotoxicity were resolved after the discontinuation of the energy drink. At high blood levels, and in some patients, imatinib may induce hepatotoxicity within the first 2 years of therapy. Thus, the patient was believed to tolerate the drug before consuming the energy drink, having used it for 7 years; however, the multicomponent nature of the energy drink raises questions on the singularity of responsibility of ginseng.

#### Grapefruit Juice

Grapefruit (*Citrus paradisi*) is not a regular herbal supplement used for medicinal purposes. As a drink, it has been well-reported to influence the pharmacokinetics of a variety of drugs when consumed together. Phytochemical constituents of grapefruit juice are potent inhibitors of CYPs and P-gp. Various comprehensive reviews have been published on the interaction between grapefruit juice (GFJ) and prescription drugs (56, 57).

In a study in 21 healthy human volunteers, concomitant intake of grapefruit juice and nilotinib caused a 60% increase in the peak concentration of nilotinib, along with a 29% increase in the AUC (18). Participants received 400 mg nilotinib with either 250 mL double strength GFJ or water in a cross-over study of two periods separated by 10-day washout period. This was attributed to the inhibitory actions of the phytochemical constituents of grapefruit juice on CYPs.

In a case report published by Valenzuela et al., a patient diagnosed with esophageal squamous cell carcinoma, had taken 250 mL of GFJ daily for more than 3 months while on docetaxel and had shown unusual pharmacokinetics of docetaxel relative to dose (17). The elimination of docetaxel had been observed to be slow in the patient, with an estimated plasma clearance of 13.2 L/h compared to the typical plasma clearance of docetaxel of 36.7 L/h. After reviewing the patient's medication records, the authors reported suspecting that GFJ might be influencing the pharmacokinetics of docetaxel in the patient. A 60% reduction in the AUC (to infinity), with a 36% increase in plasma clearance and a 10% decrease in the terminal half-life of docetaxel were observed following GFJ discontinuation. This further confirmed that GFJ suppressed the clearance of docetaxel, most likely through inhibitory activity of CYP enzymes which are responsible for the metabolism of docetaxel.

#### Milk Thistle

Milk thistle (*Silybum marianum*) is another popular herbal product used as complementary medicine in cancer patients and to boost immunity in HIV/AIDS patients. It is also used for the treatment and prevention of liver diseases. Silymarin, a mixture of biologically active flavonolignans, is the active constituent of milk thistle and generally expressed in the leaves, seeds, and fruit of the plant. Commercially available products of Milk thistle are usually provided as silymarin, a complex mixture of flavonolignans and a flavonoid. A fraction of this mixture called silibinin (containing silybin A and silybin B) have also been made commercially available. A recent publication provides a comprehensive review of these phytochemical components of Milk thistle, and their nomenclature (58). Silymarin has been clinically investigated for its anticancer activity with promising results (59, 60). Silymarin has been shown through *in vitro* studies to inhibit the activity of CYP and phase 2 enzymes (61, 62). This potential for herb-drug interaction has been shown in clinical studies, where silymarin significantly reduced the CYP2C9-mediated metabolism of losartan (63).

Due to the preponderance of use of milk thistle product among cancer patients, the potential for herb-drug interaction is a major clinical concern; however, clinical data on this is sparse. A study was conducted to determine if the inhibitory activity of milk thistle extract on CYP3A4 will translate to the alteration of the pharmacokinetics of irinotecan, a CYP3A4 substrate, in humans when taken together. The study in six cancer patients who were being treated with once-a-week irinotecan, in the course of which thrice-daily milk thistle was administered for 12 days assessed the pharmacokinetics of irinotecan and its metabolites. Authors reported that neither the short-term (4 days) nor prolonged use of milk thistle (12 days) resulted in any significant alteration in the pharmacokinetics of irinotecan. Only a slight and insignificant drop in clearance was observed with 31.2, 25.4, and 25.6 L/h in the first, second and third week, respectively, reported (19). According to the authors, potential for clinically significant interaction between silymarin and CYP3A4 substrate may not be very strong because the C_max_ of silibinin, at the usual dose, is reported to range from 0.0249 to 0.257 μM, a concentration that may be too low for CYP/P-gp inhibition (64).

This notwithstanding, in the absence of further proof, the risk of clinically significant herb-drug interaction between milk thistle and chemotherapeutic agents may still be present due to variations in silymarin concentrations in different commercially available milk thistle formulations.

#### St John's Wort

St John's wort (*Hypericum perforatum*) is a common herbal supplement widely used for the treatment of depression, anxiety, sleep disorders, and nervousness (65). Official guidelines in multiple countries have recommended St John's wort for the treatment of depression, which has increased the popularity and consumption of St John's wort among various patient groups (66). Other popular uses of St John's wort include in the treatment of premenstrual syndrome, alcohol withdrawal, and somatoform disorders (67–70). Several active phytochemical constituents including naphthodianthrones (like hypericin), phloroglucinols (like hyperforin), and flavonol glycoside (like hyperosides) have been isolated and characterized from St John's wort (71). The antidepressant activity of St John's wort has been attributed to hyperforin, the constituent with the most potent ability to inhibit the synaptic reuptake of central neurotransmitters such as dopamine, noradrenaline, and serotonin.

Several *in vitro* studies have demonstrated the ability of the extracts of St John's wort to modulate the activity of CYP and major drug transporters. For example, St John's wort has been shown as a potent inducer of CYP2B6, CYP2C19, CYP2E1, and CYP3A4. Hyperforin, in addition to its inductive effects on several CYP isoforms, is a potent inhibitor of CYP2C9 and CYP2D6. Other constituents of St John's wort have shown inhibitory activities against CYPs. For example, biapigenin, a flavonoid from St John's wort, is a potent inhibitor of CYP1A2, CYP2C9, and CYP3A4 whereas hypericin is a competitive inhibitor of CYP2C9, CYP2D6, and CYP3A4 (72). Mechanistic studies in cell lines and in animal models have demonstrated the herb-drug interaction potential of St John's wort. The effect of concomitant administration of St John's wort and several clinically important substrates of these CYPs and transporters have been investigated in humans. In some instances, clinical case reports have been published showing significant herb-drug interaction between St John's wort and prescription medicine.

In human studies and clinical case reports, St John's wort has been shown to alter the pharmacokinetics of various substrates of CYP3A4 and P-gp including omeprazole, simvastatin, cyclosporine, indinavir, verapamil, and tacrolimus (73–78).

Four clinically relevant studies retrieved from the literature, show the influence of concomitantly administered St John's wort on the pharmacokinetics of anti-cancer drugs. The influence of St John's wort on the pharmacokinetics of docetaxel, a CYP3A4 substrate, was evaluated in 10 cancer patients. Subjects were intravenously administered with 135 mg docetaxel on day 1 of the study followed by blood withdrawal and pharmacokinetic analysis. From day 7 to 21, participants were treated with commercially available 300 mg tablets of St John's wort extracts (Hyperiplant®), three-times-daily. The mean AUC∞ of docetaxel was decreased by 12%, and the total clearance increased by 14% due to the pre-supplementation with St John's wort. In addition, the C_max_ and T_1/2_ of docetaxel was decreased, non-significantly. The study also found a lower incidence of docetaxel-related adverse effects due to St John's wort supplementation (20). These observations are consistent with the mechanistic ability of St John's wort to induce CYP3A4 and accelerate the metabolism of its substrates.

In another study, the effect of St John's wort on the metabolism of irinotecan was assessed. Five cancer patients recruited for the study were treated with irinotecan with or without St John's wort supplementation for 18 days in an unblinded randomized cross-over study. St John's wort decreased the plasma level of the active metabolite, SN-38, by 42% (21). Authors also reported a mean decrease in leucocyte counts of 63% when irinotecan alone was used compared to a 4.3% decrease count when combined with St John's wort. The reduced incidence of myelosuppression was attributed to increased metabolism of irinotecan, brought about by the inductive effects of St John's wort on the metabolism of irinotecan and SN-38. The St John's wort -irinotecan combination has also been reported to mitigate against hematologic and gastrointestinal toxicities associated with irinotecan (79).

Smith and co-workers conducted an open-label cross-over study to determine the influence of St John's wort on the pharmacokinetics (PK) of imatinib in 10 healthy adult subjects (22). PK parameters were compared following a single administration of 400 mg imatinib before and after a 2-week St John's wort treatment. St John's wort reduced the median AUC of imatinib by 32%, and the observed C_max_ by 29%. This significant St John's wort -induced reduction in imatinib exposure, alongside decreased plasma half-life, occurred in all 10 participants. Additionally, the C_max_ in the presence of St John's wort was diminished in all participants but one.

In a similar study, using a 2-period design for an open-label, fixed-sequence study in 12 healthy volunteers, Frye and co-workers reported a 43% increased clearance of imatinib with a 30% reduction in imatinib exposure (23). Each of the volunteers had received 400 mg of imatinib orally on days 1 and 15, while also receiving three-times-daily 300 mg of St John's wort from days 4 to 17. Plasma imatinib were analyzed over 72 h after each imatinib administration. In addition to the increased total clearance and the reduced total exposure, St John's wort caused a 31% decrease in the plasma half-life (from 12.8 to 9 h) and a 20% decrease in the plasma C_max_ of imatinib in the subjects. All the pharmacokinetic changes were observed in all 12 participants. These effects are significant and may pose a risk for therapeutic failure in cancer patients who take St John's wort along with their therapeutic agents.

## Discussion

Despite the scarcity of data on therapeutic benefit of herbal supplements in cancer, the use of herbal products is very common among cancer patients. Studies have reported figures as high as 50–66% use of one or more complementary/alternative medicine, the majority of which are herbal preparations, concurrently with conventional cancer therapy (80, 81). This review identified six herbal products—echinacea, garlic, ginseng, grapefruit juice, milk thistle, and St John's wort—which have shown clinically relevant interactions with specific chemotherapeutic agent. Several other herbal products are commonly used among cancer patients for which there are currently no clinically relevant herb-drug interaction data, but with strong potential for interactions based on laboratory-based results. These include green tea (*Camellia sinensis*), mistletoe (*Viscum album*), evening primrose (*Oenothera paradoxa*), parsley (*Petroselinum crispum*), goldenseal (*Hydrastis canadensis*), kava (*Piper methysticum*), aloe vera (*Aloe barbadensis*), wild yam (*Dioscorea villosa*), valerian (*Valeriana officinalis*), golden root (*Rhodiola rosea*), medicinal mushrooms (including species of *Ganoderma, Grifola*, and *Trametes*), agaricus (*Agaricus campestris*), and rooibos (*Aspalathus linearis*) (82).

As highlighted earlier, herb-drug combination is particularly undesirable in cancer patients because of herb-drug interaction risks. Most herb-drug interactions are pharmacokinetic in mechanism and are brought about by either the induction or inhibition of drug-metabolizing enzymes and transport proteins. Since echinacea can inhibit and induce CYP enzymes, it is difficult to predict what effect it will have on a patient's therapy. Current data is sparse and showed conflicting outcomes as to enhancing or decreasing the effect of chemotherapy. Ginseng is another inducer that may place a patient at higher risk for adverse effects if taken along with chemotherapy. Based on the case study found, it is unclear if ginseng was the definite cause of hepatotoxicity; however, since there is evidence to suggest that ginseng induces CYP enzymes, the patient's hepatotoxicity is thought to be due to the ginseng component of the energy drink. Further studies and reports are needed to assess the interaction between ginseng and chemotherapeutic agents. By inhibiting CYP enzymes, garlic and milk thistle can effectively inhibit the metabolism of certain chemotherapeutic agents. Based on the available literature, both can clinically influence the pharmacokinetics of chemotherapeutic drugs. Interaction of grapefruit juice is unique in that most people consume grapefruit juice for non-medicinal purposes. Current literature shows that grapefruit juice caused the accumulation of CYP/P-gp substrates due to inhibition, placing patients at increased risk for adverse effects from chemotherapy. This interaction is important because it highlights the importance of diet during chemotherapy treatment.

To ensure effective care, providers should have open conversations with their patients in order to document their herb-drug use and provide necessary counseling. Patients need education on the potential beneficial and harmful effects of herbal products in cancer. Such education should include the lack of sufficient supportive data and the liberal marketing strategies employed in the sale of herbal products. Importantly, patients should understand the potential for herb-drug interaction and the attendant toxicity or therapy failure.

## Conclusion

While the beneficial effects of the commonly consumed herbal products by cancer patients is uncertain, data from human studies suggest that some of these supplements are capable of interacting with chemotherapeutic agents. It is therefore prudent and advisable to avoid the concomitant use of anti-cancer drugs and herbal products, especially echinacea, garlic, ginseng, grapefruit juice, milk thistle, and St John's wort. Clinicians and practitioners need to be vigilant in monitoring for any herb-anticancer combination.

## Author Contributions

PF and GR conceptualized the research, contributed equally to the literature search, synthesis, writing of the manuscript, and agree on the final version of the manuscript.

### Conflict of Interest

The authors declare that the research was conducted in the absence of any commercial or financial relationships that could be construed as a potential conflict of interest.

## References

[1] Rashrash M Schommer JC Brown LM. Prevalence and predictors of herbal medicine use among adults in the United States. J Patient Exp. (2017) 4:108–13. 10.1177/2374373517706612 28959715 PMC5593261

[2] McCune JS Hatfield AJ Blackburn AA Leith PO Livingston RB Ellis GK. Potential of chemotherapy–herb interactions in adult cancer patients. Support Care Cancer. (2004) 12:454–62. 10.1007/s00520-004-0598-1 14991387

[3] Luo Q Asher GN. Use of dietary supplements at a comprehensive cancer center. J Altern Complement Med. (2018) 24:981–7. 10.1089/acm.2018.0183 30247972

[4] Bailey DG Spence JD Munoz C Arnold JM. Interaction of citrus juices with felodipine and nifedipine. Lancet. (1991) 337:268–9. 10.1016/0140-6736(91)90872-M 1671113

[5] Burnham BE. Garlic as a possible risk for postoperative bleeding. Plast Reconstr Surg. (1995) 95:213. 10.1097/00006534-199501000-00060 7809259

[6] Huang Z Xiao B Wang X Li Y Deng H. Betel nut indulgence as a cause of epilepsy. Seizure. (2003) 12:406–8. 10.1016/S1059-1311(02)00377-1 12915088

[7] Engelberg D McCutcheon A Wiseman S. A case of ginseng-induced mania. J Clin Psychopharmacol. (2001) 21:535–7. 10.1097/00004714-200110000-00015 11593083

[8] Alnaim L. Therapeutic drug monitoring of cancer chemotherapy. J Oncol Pharm Pract. (2007) 13:207–21. 10.1177/1078155207081133 18045780

[9] Gieschke R Burger HU Reigner B Blesch KS Steimer JL. Population pharmacokinetics and concentration–effect relationships of capecitabine metabolites in colorectal cancer patients. Br J Clin Pharmacol. (2003) 55:252–63. 10.1046/j.1365-2125.2003.01765.x 12630975 PMC1884209

[10] Phan VH Moore MM McLachlan AJ Piquette-Miller M Xu H Clarke SJ. Ethnic differences in drug metabolism and toxicity from chemotherapy. Expert Opin Drug Metab Toxicol. (2009) 5:243–57. 10.1517/17425250902800153 19331590

[11] Fattinger K Roos M Vergeres P Holenstein C Kind B Masche U Epidemiology of drug exposure and adverse drug reactions in two Swiss departments of internal medicine. Br J Clin Pharmacol. (2000) 499:158–67. 10.1046/j.1365-2125.2000.00132.x PMC2014906 10671911

[12] Zoppi M Braunschweig S Kuenzi UP Maibach R Hoigné R. Incidence of lethal adverse drug reactions in the comprehensive hospital drug monitoring, a 20-year survey, 1974–1993, based on the data of Berne/St Gallen. Eur J Clin Pharmacol. (2000) 56:427–30. 10.1007/s002280000158 11009053

[13] Bossaer JB Odle BL. Probable etoposide interaction with Echinacea. J Diet Suppl. (2012) 9:90–5. 10.3109/19390211.2012.682643 22607644

[14] Goey AK Meijerman I Rosing H Burgers JA Mergui-Roelvink M Keessen M. The effect of Echinacea purpurea on the pharmacokinetics of docetaxel. Br J Clin Pharmacol. (2013) 76:467–74. 10.1111/bcp.12159 23701184 PMC3769673

[15] Cox MC Low J Lee J Walshe J Denduluri N Berman A. Influence of garlic (*Allium sativum*) on the pharmacokinetics of docetaxel. Clin Cancer Res. (2006) 12:4636–40. 10.1158/1078-0432.CCR-06-0388 16899612

[16] Bilgi N Bell K Ananthakrishnan AN Atallah E. Imatinib and Panax ginseng: a potential interaction resulting in liver toxicity. Ann Pharmacother. (2010) 44:926–8. 10.1345/aph.1M715 20332334

[17] Valenzuela B Rebollo J Pérez T Brugarolas A Pérez-Ruixo JJ. Effect of grapefruit juice on the pharmacokinetics of docetaxel in cancer patients: a case report. Br J Clin Pharmacol. (2011) 72:978. 10.1111/j.1365-2125.2011.04052.x 21692829 PMC3244646

[18] Yin OQ Gallagher N Li A Zhou W Harrell R Schran H. Effect of grapefruit juice on the pharmacokinetics of nilotinib in healthy participants. J Clin Pharmacol. (2010) 50:188–94. 10.1177/0091270009336137 19948946

[19] Van Erp NP Baker SD Zhao M Rudek MA Guchelaar HJ Nortier JW. Effect of milk thistle (Silybum marianum) on the pharmacokinetics of irinotecan. Clin Cancer Res. (2005) 11:7800–6. 10.1158/1078-0432.CCR-05-1288 16278402

[20] Goey AK Meijerman I Rosing H Marchetti S Mergui-Roelvink M Keessen M. The effect of St John's wort on the pharmacokinetics of docetaxel. Clin Pharmacokinet. (2014) 53:103–10. 10.1007/s40262-013-0102-5 24068654

[21] Mathijssen RH Verweij J de Bruijn P Loos WJ Sparreboom A. Effects of St. John's wort on irinotecan metabolism. J Nat Cancer Inst. (2002) 94:1247–9. 10.1093/jnci/94.16.1247 12189228

[22] Smith P Bullock JM Booker BM Haas CE Berenson CS Jusko WJ. The influence of St. John's wort on the pharmacokinetics and protein binding of imatinib mesylate. Pharmacotherapy. (2004) 24:1508–14. 10.1592/phco.24.16.1508.50958 15537555

[23] Frye RF Fitzgerald SM Lagattuta TF Hruska MW Egorin MJ. Effect of St John's wort on imatinib mesylate pharmacokinetics. Clin Pharmacol Ther. (2004) 76:323–9. 10.1016/j.clpt.2004.06.007 15470331

[24] Fasinu P Bouic P Rosenkranz B. An overview of the evidence and mechanisms of herb–drug interactions. Front Pharmacol. (2012) 3:69. 10.3389/fphar.2012.00069 22557968 PMC3339338

[25] Izzo AA. Herb–drug interactions: an overview of the clinical evidence. Fund Clin Pharmacol. (2005) 19:1–6. 10.1111/j.1472-8206.2004.00301.x 15660956

[26] Sheweita SA Newairy AA Mansour HA Yousef MI. Effect of some hypoglycemic herbs on the activity of phase I and II drug-metabolizing enzymes in alloxan-induced diabetic rats. Toxicology. (2002) 174:131–9. 10.1016/S0300-483X(02)00048-3 11985890

[27] Iqbal M Sharma SD Okazaki Y Fujisawa M Okada S. Dietary supplementation of curcumin enhances antioxidant and phase II metabolizing enzymes in ddY male mice: possible role in protection against chemical carcinogenesis and toxicity. Pharmacol Toxicol. (2003) 92:33–8. 10.1034/j.1600-0773.2003.920106.x 12710595

[28] Mohamed ME Frye RF. Inhibition of intestinal and hepatic glucuronidation of mycophenolic acid by Ginkgo biloba extract and flavonoids. Drug Metab Dispos. (2010) 38:270–5. 10.1124/dmd.109.030080 19889883

[29] Mohamed ME Frye RF. Inhibitory effects of commonly used herbal extracts on UDP-glucuronosyltransferase 1A4, 1A6, and 1A9 enzyme activities. Drug Metab Dispos. (2011) 39:1522–8. 10.1124/dmd.111.039602 21632963 PMC3164271

[30] Juliano RL Ling V. A surface glycoprotein modulating drug permeability in Chinese hamster ovary cell mutants. Biochim Biophys Acta. (1976) 455:152–62. 10.1016/0005-2736(76)90160-7 990323

[31] Zhang S Morris ME. Effects of the flavonoids biochanin A, morin, phloretin, and silymarin on P-glycoprotein-mediated transport. J Pharmacol Exp Ther. (2003) 304:1258–67. 10.1124/jpet.102.044412 12604704

[32] Choi CH Kang G Min YD. Reversal of P-glycoprotein-mediated multidrug resistance by protopanaxatriol ginsenosides from Korean red ginseng. Planta Med. (2003) 69:235–40. 10.1055/s-2003-38483 12677527

[33] Bhardwaj RK Glaeser H Becquemont L Klotz U Gupta SK Fromm MF. Piperine, a major constituent of black pepper, inhibits human P-glycoprotein and CYP3A4. J Pharmacol Exp Ther. (2002) 302:645–50. 10.1124/jpet.102.034728 12130727

[34] Nabekura T Kamiyama S Kitagawa S. Effects of dietary chemopreventive phytochemicals on P-glycoprotein function. Biochem Biophys Res Commun. (2005) 327:866–70. 10.1016/j.bbrc.2004.12.081 15649425

[35] Block KI Mead MN. Immune system effects of echinacea, ginseng, and astragalus: a review. Integr Cancer Therapies. (2003) 2:247–67. 10.1177/1534735403256419 15035888

[36] Sultan MT Buttxs MS Qayyum MM Suleria HA. Immunity: plants as effective mediators. Crit Rev Food Sci Nutr. (2014) 54:1298–308. 10.1080/10408398.2011.633249 24564587

[37] Miller SC. Echinacea: a miracle herb against aging and cancer? Evidence *in vivo* in mice. Evid Based Complement Alternat Med. (2005) 2:309–14. 10.1093/ecam/neh118 16136209 PMC1193558

[38] Binns CW Lee MK Lee AH. Problems and prospects: public health regulation of dietary supplements. Annu Rev Public Health. (2018) 39:403–20. 10.1146/annurev-publhealth-040617-013638. 29272167

[39] Awortwe C Manda VK Avonto C Khan SI Khan IA Walker LA. Echinacea purpurea up-regulates CYP1A2, CYP3A4 and MDR1 gene expression by activation of pregnane X receptor pathway. Xenobiotica. (2015) 45:218–29. 10.3109/00498254.2014.973930 25377539 PMC4355449

[40] Hansen TS Nilsen OG. *In vitro* CYP3A4 metabolism: inhibition by Echinacea purpurea and choice of substrate for the evaluation of herbal inhibition. Basic Clin Pharmacol Toxicol. (2008) 103:445–9. 10.1111/j.1742-7843.2008.00307.x 18947363

[41] Gorski JC Huang SM Pinto A Hamman MA Hilligoss JK Zaheer NA. The effect of echinacea (Echinacea purpurea root) on cytochrome P450 activity *in vivo*. Clin Pharmacol Ther. (2004) 75:89–100. 10.1016/j.clpt.2003.09.013 14749695

[42] Moltó J Valle M Miranda C Cedeño S Negredo E Barbanoj MJ. Herb-drug interaction between Echinacea purpurea and darunavir-ritonavir in HIV-infected patients. Antimicrob Agents Chemother. (2011) 55:326–30. 10.1128/AAC.01082-10 21078942 PMC3019656

[43] Ernst E Pittler MH Wider B The Desktop Guide to Complementary and Alternative Medicine. Philadelphia, PA: Mosby Elsevier (2006).

[44] Foster BC Gallicano K Cameron W Choudhri SH Constituents of garlic inhibit cytochrome P450 3A4-mediated drug metabolism. Can J Infect Dis. (1998) 9(Suppl A):472P.

[45] Gillis CN. Panax ginseng pharmacology: a nitric oxide link? Biochem Pharmacol. (1997) 54: 1–8. 10.1016/S0006-2952(97)00193-7 9296344

[46] Chen X. Cardiovascular protection by ginsenosides and their nitric oxide releasing action. Clin Exp Pharmacol Physiol. (1996) 23:728–32. 10.1111/j.1440-1681.1996.tb01767.x 8886498

[47] Yun YS Moon HS Oh YR Jo SK Kim YJ Yun TK. Effect of red ginseng on natural killer cell activity in mice with lung adenoma induced by urethan and benzo(a)pyrene. Cancer Detection Prev Suppl. (1987) 1:301–9. 3480057

[48] Yuan CS Attele AS Wu JA Liu D Modulation of American ginseng on brainstem GABAergic effects in the rat. J Ethnopharmacol. (1998) 63:215–22. 10.1016/S0378-8741(98)00066-X 9849631

[49] Zhu JH Takeshita T Kitagawa I Morimoto K. Suppression of the formation of sister chromatid exchanges by low concentrations of ginsenoside Rh2 in human blood lymphocytes Cancer Res. (1995) 55:1221–3. 7882311

[50] Zheng YF Bae SH Choi EJ Park JB Kim SO Jang MJ. Evaluation of the *in vitro/in vivo* drug interaction potential of BST204, a purified dry extract of ginseng, and its four bioactive ginsenosides through cytochrome P450 inhibition/induction and UDP-glucuronosyltransferase inhibition. Food Chem Toxicol. (2014) 68:117–27. 10.1016/j.fct.2014.03.004 24632066

[51] Liu KH Kim MJ Jeon BH Shon JH Cha IJ Cho KH. Inhibition of human cytochrome P450 isoforms and NADPH-CYP reductase *In vitro* by 15 herbal medicines, including Epimedii herba. J Clin Pharm Ther. (2006) 31:83–91. 10.1111/j.1365-2710.2006.00706.x 16476124

[52] Henderson GL Harkey MR Gershwin ME Hackman RM Stern JS Stresser DM. Effects of ginseng components on c-DNA-expressed cytochrome P450 enzyme catalytic activity. Life Sci. (1999) 65:PL209–14. 10.1016/S0024-3205(99)00407-5 10574228

[53] Gum SI Jo SJ Ahn SH Kim SG Kim JT Shin HM The potent protective effect of wild ginseng (Panax ginseng CA Meyer) against benzo [α] pyrene-induced toxicity through metabolic regulation of CYP1A1 and GSTs. J Ethnopharmacol. (2007) 112:568–76. 10.1016/j.jep.2007.05.014 17590295

[54] Kim HJ Chun YJ Park JD Kim SI Roh JK Jeong TC. Protection of rat liver microsomes against carbon tetrachloride-induced lipid peroxidation by red ginseng saponin through cytochrome P450 inhibition. Planta Medica. (1997) 63:415–8. 10.1055/s-2006-957724 9342944

[55] Malati CY Robertson SM Hunt JD Chairez C Alfaro RM Kovacs JA. Influence of Panax ginseng on Cytochrome P450 (CYP) 3A and P-glycoprotein (P-gp) activity in healthy participants. J Clin Pharmacol. (2012) 52:932–9. 10.1177/0091270011407194 21646440 PMC3523324

[56] Dahan A Altman H. Food–drug interaction: grapefruit juice augments drug bioavailability—mechanism, extent and relevance. Eur J Clin Nutr. (2004) 58:1. 10.1038/sj.ejcn.1601736 14679360

[57] Chen M Zhou SY Fabriaga E Zhang PH Zhou Q. Food-drug interactions precipitated by fruit juices other than grapefruit juice: an update review. J Food Drug Anal. (2018) 26:S61–71. 10.1016/j.jfda.2018.01.009 29703387 PMC9326888

[58] Kroll DJ Shaw HS Oberlies NH. Milk thistle nomenclature: why it matters in cancer research and pharmacokinetic studies. Integr Cancer Ther. (2007) 6:110–9. 10.1177/1534735407301825 17548790

[59] Schroder FH Roobol MJ Boeve ER de Mutsert R Zuijdgeest-van Leeuwen SD Kersten I. Randomized, double-blind, placebo-controlled crossover study in men with prostate cancer and rising PSA: effectiveness of a dietary supplement. Eur Urol. (2005) 48:922–30. 10.1016/j.eururo.2005.08.005 16263208

[60] Hoh C Boocock D Marczylo T Singh R Berry DP Dennison AR. Pilot study of oral silibinin, a putative chemopreventive agent, in colorectal cancer patients: silibinin levels in plasma, colorectum, and liver and their pharmacodynamic consequences. Clin Cancer Res. (2006) 12:2944–50. 10.1158/1078-0432.CCR-05-2724 16675592

[61] Brantley SJ Oberlies NH Kroll DJ Paine MF. Two flavonolignans from milk thistle (Silybum marianum) inhibit CYP2C9-mediated warfarin metabolism at clinically achievable concentrations. J Pharmacol Exp Ther. (2010) 332:1081–7. 10.1124/jpet.109.161927 19934397 PMC2835426

[62] Venkataramanan R Ramachandran V Komoroski BJ Zhang S Schiff PL Strom SC. Milk thistle, a herbal supplement, decreases the activity of CYP3A4 and uridine diphosphoglucuronosyl transferase in human hepatocyte cultures. Drug Metab Dispos. (2000) 28:1270–3. 11038151

[63] Han Y Guo D Chen Y Chen Y Tan ZR Zhou HH. Effect of silymarin on the pharmacokinetics of losartan and its active metabolite E-3174 in healthy Chinese volunteers. Eur J Clin Pharmacol. (2009) 65:585–91. 10.1007/s00228-009-0624-9 19221727

[64] Wen Z Dumas TE Schrieber SJ. Pharmacokinetics and metabolic profile of free, conjugated, and total silymarin flavonolignans in human plasma after oral administration of milk thistle extract. Drug Metab Dispos. (2008) 36:65–72. 10.1124/dmd.107.017566 17913795

[65] Linde K Ramirez G Mulrow CD Pauls A Weidenhammer W Melchart D. St John's wort for depression—an overview and meta-analysis of randomised clinical trials. BMJ. (1996) 313:253–8. 10.1136/bmj.313.7052.253 8704532 PMC2351679

[66] Williams JW Jr Mulrow CD Chiquette E Noël PH Aguilar C Cornell J. A systematic review of newer pharmacotherapies for depression in adults: evidence report summary. Ann Intern Med. (2000) 132:743–56. 10.7326/0003-4819-132-9-200005020-00011 10787370

[67] Stevinson C Ernst E. A pilot study of Hypericum perforatum for the treatment of premenstrual syndrome. Br J Obstet Gynaecol. (2000) 107:870–6. 10.1111/j.1471-0528.2000.tb11085.x 10901558

[68] Winkel R Koritsch HD Piayda H St John's wort extract LI 160 in depressive, alcohol-addicted patients [abstract]. Phytomedicine. (2000) 7(Suppl 2):19.

[69] Müller T Mannel M Murck H Rahlfs VW. Treatment of somatoform disorders with St John's wort: a randomized, double-blind and placebo-controlled trial. Psychosom Med. (2004) 66: 538–47. 10.1097/01.psy.0000128900.13711.5b 15272100

[70] Volz HP Murck H Kasper S Möller HJ. St John's wort extract (LI 160) in somatoform disorders: results of a placebo-controlled trial. Psychopharmacology. (2002) 164:294–300. 10.1007/s00213-002-1171-6 12424553

[71] Greeson JM Sanford B Monti DA. St John's wort (Hypericum perforatum): a review of the current pharmacological, toxicological, and clinical literature. Psychopharmacology. (2001) 153:402–14. 10.1007/s002130000625 11243487

[72] Obach RS. Inhibition of human cytochrome P450 enzymes by constituents of St John's Wort, an herbal preparation used in the treatment of depression. J Pharmacol Exp Ther. (2000) 294:88–95. 10871299

[73] Wang LS Zhou G Zhu B Wu J Wang JG Abd El-Aty AM. St John's wort induces both cytochrome P450 3A4-catalyzed sulfoxidation and 2C19-dependent hydroxylation of omeprazole. Clin Pharmacol Ther. (2004) 75:191–7. 10.1016/j.clpt.2003.09.014 15001970

[74] Sugimoto K Ohmori M Tsuruoka S Nishiki K Kawaguchi A Harada K. Different effects of St John's wort on the pharmacokinetics of simvastatin and pravastatin. Clin Pharmacol Ther. (2001) 70:518–24. 10.1016/S0009-9236(01)64092-X 11753267

[75] Bauer S Stormer E Johne A Krüger H Budde K Neumayer HH Alterations in cyclosporine A pharmacokinetics and metabolism during treatment with St John's wort in renal transplant patients. Br J Clin Pharmacol. (2003) 55:203–11. 10.1046/j.1365-2125.2003.01759.x 12580993 PMC1894728

[76] Piscitelli SC Burstein AH Chaitt D Alfaro RM Falloon J. Indinavir concentrations and St John's wort. Lancet. (2000) 355:547–8. 10.1016/S0140-6736(99)05712-8 10683007

[77] Tannergren C Engman H Knutson L Hedeland M Bondesson U Lennernäs H. St John's wort decreases the bioavailability of R- and S-verapamil through induction of the first-pass metabolism. Clin Pharmacol Ther. (2004) 75:298–309. 10.1016/S0009-9236(03)00773-2 15060508

[78] Mai I Stormer E Bauer S Krüger H Budde K Roots I. Impact of St John's wort treatment on the pharmacokinetics of tacrolimus and mycophenolic acid in renal transplant patients. Nephrol Dial Transplant. (2003) 18:819–22. 10.1093/ndt/gfg002 12637655

[79] Hu ZP Yang XX Chan SY Xu AL Duan W Zhu YZ. St. John's wort attenuates irinotecan-induced diarrhea via down-regulation of intestinal pro-inflammatory cytokines and inhibition of intestinal epithelial apoptosis. Toxicol Appl Pharmacol. (2006) 216:225–37. 10.1016/j.taap.2006.05.020 17015070

[80] Henderson JW Donatelle RJ. Complementary and alternative medicine use by women after completion of allopathic treatment for breast cancer. Altern Ther Health Med. (2004) 10:52–7. 14727500

[81] Zeller T Muenstedt K Stoll C Schweder J Senf B Ruckhaeberle E. Potential interactions of complementary and alternative medicine with cancer therapy in outpatients with gynecological cancer in a comprehensive cancer center. J Cancer Res Clin Oncol. (2013) 139:357–65. 10.1007/s00432-012-1336-6 23099993 PMC11824351

[82] Alsanad SM Williamson EM Howard RL. Cancer patients at risk of herb/food supplement–drug interactions: a systematic review. Phytother Res. (2014) 28:1749–55. 10.1002/ptr.5213 25158128

